# Prenatal diagnosis of a variant of the azygos venous system

**DOI:** 10.1186/s40064-016-2956-0

**Published:** 2016-08-11

**Authors:** Clelia Lo Verso, Valentina Cigna, Gianfranca Damiani, Laura Lo Verso, Rossella Conti, Vincenzo Duca

**Affiliations:** 1 Department of Sciences for Health Promotion and Mother and Child Care, Palermo University, Palermo, Italy; 2 U.O.S. Fetal Medicine and Prenatal Diagnosis, A.O. Hospital Villa Sofia-Cervello , Palermo, Italy; 3 U.O. of Neonatology and NICU, A.O. Hospital Villa Sofia-Cervello, Palermo, Italy

**Keywords:** Azygos vein, Hemiazygos vein, Accessory hemiazygos vein, Variants, Prenatal diagnosis

## Abstract

**Background:**

The azygos venous system consists of the azygos vein on the right side and the hemiazygos and accessory hemiazygos on the left side. The azygos vein runs through the abdominal cavity along the right side of the vertebral bodies, in a cranial direction, passes through the diaphragm and reaches the mediastinum, where it forms the arch of the azygos which flows into the superior vena cava. Along its course, the azygos vein communicates with the intercostal veins on the right, the hemiazygos vein that collects blood from the left lower intercostal veins, and accessory hemiazygos vein that drains into the left upper intercostal veins. The last two, at the level of the seventh thoracic vertebra, unite and end in the azygos vein. The accessory hemiazygos vein is normally included in the length between T4 and T8. The embryological origin of the accessory hemiazygos vein is the result of an expansion in the direction of the cranial hemiazygos vein, which comes from the left upper sovracardinale vein (Dudiak et al. in Semin Roentgenol 24(1):47–55, 1989; Radiographics 11(2):233–246, 1991; Webb et al. in Am J Roentgenol 139(1):157–161, 1982).

**Findings:**

This case report describes a rare variant of azygos vein system identified in prenatal diagnosis and confirmed by postnatal ultrasonography.

**Conclusions:**

The observation of the patient has excluded hemodynamic alterations associated with vascular anomaly.

## Findings

**Fig. 1:**
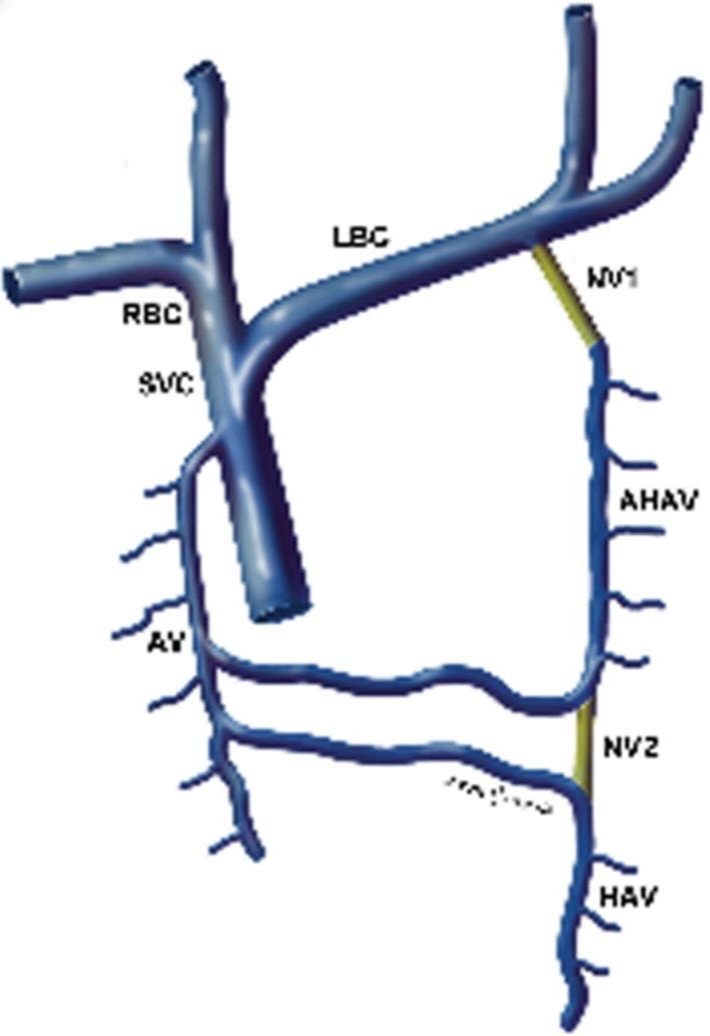
Reconstruction of the anatomical variant of azygos vein system identified in this patient: the hemiazygos and accessory hemiazygos veins form common channels which drain into the brachiocephalic vein

**Fig. 2:**
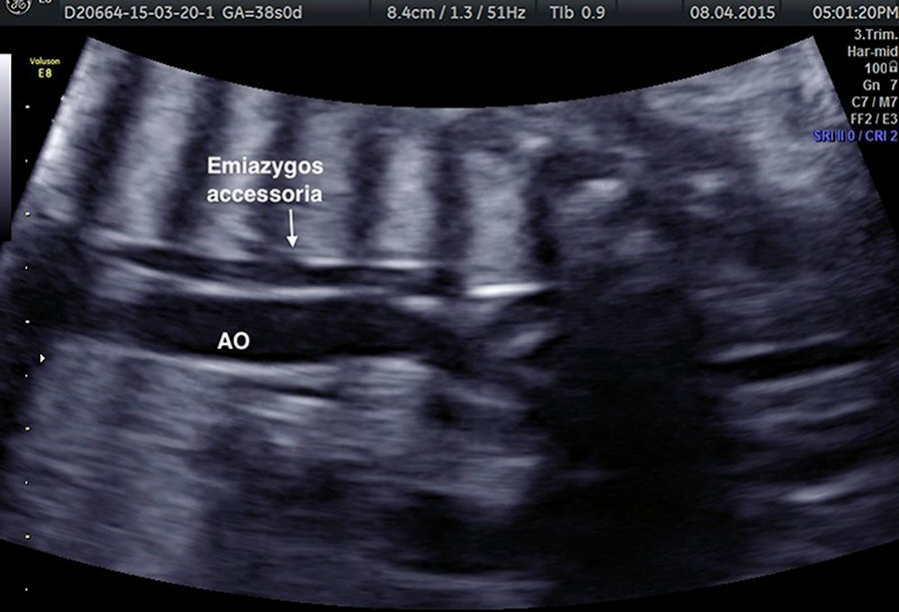
The prenatal ultrasound shows a vessel with parallel course on the left and posteriorly to the thoracic aorta

**Fig. 3:**
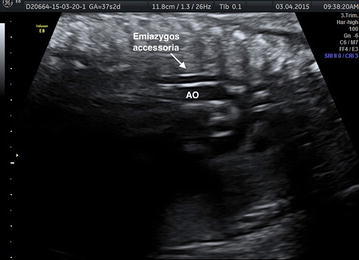
The prenatal ultrasound shows the accessory hemiazygos that drain into the left venous trunk brachial-cephalic

Our study has focused on a 27-year-old woman, in first pregnancy at a gestational age of 38 weeks, who has been submitted to fetal echocardiography. During the investigation it has been identified the presence of a vessel with parallel course, rear and left to thoracic aorta (Fig. 2). The prenatal ultrasonography with color Doppler has showed a hemiazygos accessory vein that, from the diaphragmatic dome, joining with the superior intercostal vein, seemed to drain into the venous trunk brachial-cephalic left (Fig. 3). We have assumed it was a rare variant of the azygos vein system: the hemiazygos and accessory hemiazygos veins form common channels which drain into the brachiocephalic vein (Fig. 1) (Blackmon and Franco 2011). The accessory hemiazygos vein, also called the superior hemiazygos vein, drains into the superior left hemithorax. In the majority of cases there is a small connection to the left superior intercostal vein, and rarely, 1–2 % of the time, the accessory hemiazygos vein drains into the brachiocephalic vein (Galwa et al. 2007).

**Fig. 4:**
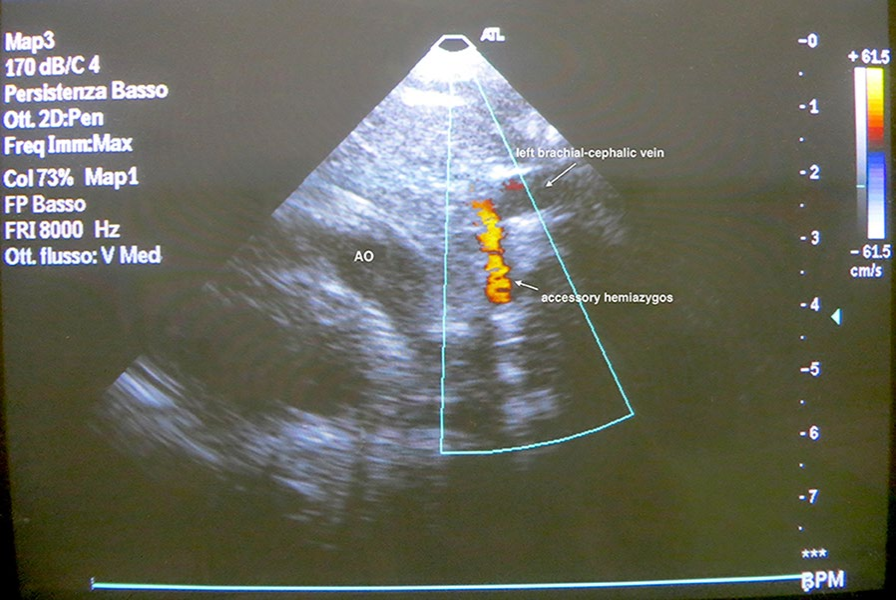
The postnatal echocardiography, through color—Doppler, shows the anterograde flow of blood draining in the left brachiocephalic trunk, as reconstructed in anatomical model in Fig. 1

We have started a follow-up with prenatal echocardiography. The patient was born by caesarean section at 40 + 4 w.g, weight 3280 g, stature 50 cm, CC 34 cm, Apgar 1′:9, 5′:10. He has had a good adaptation to extrauterine life. Postnatal echocardiography has confirmed the presence of a hemiazygos accessory vein that drains into the left brachial-cephalic trunk (Fig. 4). The ultrasound examination has showed no other anatomical or functional cardiac abnormalities. Through color—Doppler it has been possible to confirm the absence of hemodynamic changes in accordance with following the vascular variant.

## Conclusions

This vascular anomaly has never been detected prenatally. The clinical case we have described is the ideal diagnostic procedure to be followed in a patient with vascular malformation. The prenatal recognition of the vascular anatomy has allowed us to monitor the patient since fetal life and to orient the mode of delivery and assistance at birth. The prenatal ultrasound follow-up has been crucial to monitor the heart function of the fetus, as it has excluded conditions of volume overload and pressure overload of the heart chambers, and to see any other cardiac malformations. We have excluded that this variant of the azygos venous system represents an indication to early cesarean section. Finally, the postnatal ultrasound follow-up has confirmed that this anomaly does not cause hemodynamic vascular changes and does not expose the patient to other cardiovascular complications.

In the management of this case report, it has been very important the collaboration between gynecologists and neonatologists who have accompanied the mother and her unborn child with prudence and expertise providing them with the best care.

## Abbreviations

IVC: inferior vena cava
w.g.: weeks of gestation
CC: head circumference
